# Integrated *In Vivo* Genotoxicity Assessment of Procarbazine Hydrochloride Demonstrates Induction of *Pig‐a* and *LacZ* Mutations, and Micronuclei, in MutaMouse Hematopoietic Cells

**DOI:** 10.1002/em.22271

**Published:** 2019-01-18

**Authors:** Clotilde Maurice, Stephen D. Dertinger, Carole L. Yauk, Francesco Marchetti

**Affiliations:** ^1^ Environmental Health Science and Research Bureau Health Canada Ottawa Ontario Canada; ^2^ Litron Laboratories Rochester New York

**Keywords:** mutagenicity, bone marrow, red blood cells, mutation, micronuclei

## Abstract

Procarbazine hydrochloride (PCH) is a DNA‐reactive hematopoietic carcinogen with potent and well‐characterized clastogenic activity. However, there is a paucity of *in vivo* mutagenesis data for PCH, and *in vitro* assays often fail to detect the genotoxic effects of PCH due to the complexity of its metabolic activation. We comprehensively evaluated the *in vivo* genotoxicity of PCH on hematopoietic cells of male MutaMouse transgenic rodents using a study design that facilitated assessments of micronuclei and *Pig‐a* mutation in circulating erythrocytes, and *lacZ* mutant frequencies in bone marrow. Mice were orally exposed to PCH (0, 6.25, 12.5, and 25 mg/kg/day) for 28 consecutive days. Blood samples collected 2 days after cessation of treatment exhibited significant dose‐related induction of micronuclei in both immature and mature erythrocytes. Bone marrow and blood collected 3 and 70 days after cessation of treatment also showed significantly elevated mutant frequencies in both the *lacZ* and *Pig‐a* assays even at the lowest dose tested. PCH‐induced *lacZ* and *Pig‐a* (immature and mature erythrocytes) mutant frequencies were highly correlated, with R^2^ values ≥0.956, with the exception of *lacZ vs. Pig*‐*a* mutants in mature erythrocytes at the 70‐day time point (R^2^ = 0.902). These results show that PCH is genotoxic *in vivo* and demonstrate that the complex metabolism and resulting genotoxicity of PCH is best evaluated in intact animal models. Our results further support the concept that multiple biomarkers of genotoxicity, especially hematopoietic cell genotoxicity, can be readily combined into one study provided that adequate attention is given to manifestation times. Environ. Mol. Mutagen. 60:505–512, 2019. © 2018 Her Majesty the Queen in Right of Canada

## INTRODUCTION

Procarbazine hydrochloride (PCH) is a hydrazine derivative that is reemerging as a useful drug that in combination with other anti‐neoplastic agents is effective at treating several malignancies, especially gliomas and Hodgkin's lymphoma (Armand et al. 2007). While clearly efficacious, concerns about PCH treatment‐induced tumors exist (Levine and Bloomfield 1992). Mouse bioassays indicate that lung and hematopoietic cells are important target sites (IARC 1981). Furthermore, cancer patients treated with PCH in combination with other antineoplastic agents have an elevated risk of secondary malignancies, especially hematopoietic cancers (IARC 1981; Kaldor et al. 1988).

PCH appears to be a particularly effective *in vivo* clastogen inducing both micronuclei in bone marrow (Romagna and Schneider 1990) and DNA breaks as detected by the alkaline elution (Holme et al. 1989) and comet (Sasaki et al. 1998) assays in multiple tissues including stomach and liver. Furthermore, significant increases in median percent tail DNA were recently reported in bone marrow, liver, kidney, and lungs of rats, 1 day after a 28‐day exposure to 30 or 60 mg PCH/kg/day (Chen et al. 2019). However, PCH's mutagenic activity is less well‐characterized. For instance, using the transgenic MutaMouse model, Suzuki et al. (1999) showed that a single intraperitoneal (i.p.) injection of 50 mg PCH/kg was a potent micronucleus inducer in blood (mean fold change: 19.6x), while no change to *lacZ* mutation frequencies were observed in multiple tissues. In contrast, PCH mutagenicity was evident in lung, bone marrow, and spleen after five daily administrations of 150 mg/kg by i.p. injection (Suzuki et al. 1999). Following a 3‐day exposure regimen by oral gavage Phonethepswath et al. (2013) confirmed the strong clastogenic activity of PCH and observed a moderate but variable mutagenic response using the rodent phosphatidylinositol glycan‐class A (*Pig‐a*) assay. Together with the rodent bioassay data and occurrences of therapy‐related tumors noted above, the weight of evidence provided by *in vivo* data implies that PCH is a carcinogen with a genotoxic mode of action that clearly involves clastogenicity, with some evidence of mutagenicity as well. Based on these findings, the mutagenic potential of PCH deserves more analysis and consideration, including an assessment of the impact of longer exposure duration, different sampling times, and a comparison of different genetic endpoints within the same experimental system.

PCH undergoes complex metabolic activation and forms intermediates that have the capacity to react with DNA. One presumably important intermediate is the methyl diazonium ion that induces *O* ^6^‐methyl‐guanine adducts (Souliotis et al. 1994; Pletsa et al. 1997). The complexity of PCH metabolism is illustrated by the fact that a free radical, a reactive aldehyde, and an arylmethyl diazonium ion have also been implicated as significant contributors to observed toxic effects (IARC 1981; Prough and Tweedie 1987; Suzuki et al. 1999). Given the reactivity of these various metabolites, the well‐documented *in vivo* genotoxicity of the drug is not surprising (IARC 1981). At the same time, the complex metabolism of PCH coupled with deficiencies with standard exogenous metabolic activation systems is considered responsible for the high occurrence of false negative *in vitro* results (reviewed in Suzuki et al. 1999). Indeed, bacterial mutagenicity data are generally negative (McCann et al. 1975; Bronzetti et al. 1979). Mutagenic effects were observed in V79 cells (Suter 1987), but the clearest effects required nonstandard metabolic activation systems. There are some reports of positive mouse lymphoma assay data with the resultant colonies being described as small, suggesting a clastogenic mode of action (Clive et al. 1988). However, Vian et al. (1993) failed to induce micronuclei in cultures of PCH‐treated primary human lymphocytes, with or without rat liver S9. Thus, PCH represents an example where the genotoxic potential is best evaluated *in vivo*.

There is growing interest in integrating various testing approaches to significantly improve the manner in which genotoxicity testing is accomplished and reducing the number of animals required for testing. These integrated approaches provide a mechanism to precisely quantify different genotoxic endpoints within the same experimental design and identify which endpoints are most relevant to adverse effects in humans. We therefore considered PCH to be an interesting test case for evaluating the merits of an integrated study design that would allow us to comprehensively assess hematopoietic cell genotoxicity, both for chromosomal damage and gene mutation potential. The treatment period consisted of 28 consecutive days of exposure, and evaluation of chromosomal damage was accomplished by measuring micronucleus frequencies in both immature and mature peripheral blood erythrocytes (MacGregor et al. 1980). We used the MutaMouse transgenic mouse model that harbor ~29 tandem copies of a recombinant λgt10 phage vector containing a mutation‐reporting *Escherichia coli lacZ* gene on each copy of chromosome 3 (Shwed et al. 2010) and facilitated assessments of mutations in both the *lacZ* transgene (Gingerich et al. 2014) and the endogenous *Pig‐a* gene (Gollapudi et al. 2015). We quantified mutations arising in these two reporter genes at two time points: (1) 3 days from the end of the last treatment as recommended in the Organisation of Economic Co‐operation and Development (OECD) test guideline 488 (OECD 2013); and (2) 70 days after cessation of treatment to evaluate effects in long‐lived progenitor cells. The results are discussed in terms of the high information content provided by this multiple endpoint study design, including comparison of mutation frequencies at two gene loci, one endogenous and one transgenic, and chromosomal damage.

## MATERIALS AND METHODS

### Reagents and Other Supplies

PCH (CAS no. 36670‐1; purity ≥98%) was purchased from Sigma‐Aldrich, Oakville, ON, Canada. Lympholyte®‐Mammal cell separation reagent was purchased from CedarLane, Burlington, NC. Anti‐PE MicroBeads, LS Columns, and a QuadroMACS™ separator were from Miltenyi Biotec, Bergisch Gladbach, Germany. CountBright™ absolute count beads and fetal bovine serum (FBS) were purchased from Invitrogen, Carlsbad, CA. Anticoagulant solution, buffered salt solution, nucleic acid dye solution (contains SYTO® 13), anti‐CD24‐PE, and anti‐CD61‐PE were from Mouse Blood *In Vivo* MutaFlow® Kits (Litron Laboratories, Rochester, NY). Hank's balanced salt solution (HBSS) was purchased from MediaTech, Herndon, VA. Reagents used for flow cytometric micronucleus scoring (anticoagulant solution, buffered salt solution, stock propidium iodide solution, anti‐CD71‐FITC, and anti‐CD61‐PE solutions, stock RNase solution, and malaria biostandards) were from *In Vivo* Mouse MicroFlow® Kits (Litron Laboratories).

For the *lacZ* assay, phenyl‐beta‐D‐galactopyranoside (P‐Gal) was purchased from G‐Biosciences, St Louis, MO; proteinase K was from Invitrogen, Burlington, ON, Canada; packaging extract kits were from Agilent Technologies, Mississauga, ON, Canada; all other chemicals were purchased from Sigma‐Aldrich.

### Animals, Treatments, Blood Harvests

Experiments were conducted with the oversight of the Health Canada Ottawa Animal Care Committee, which approved all animal procedures. Male MutaMouse rodents were obtained from a colony maintained at Health Canada. Mice were allowed to acclimate for approximately 10 days, and their age at the start of the treatment was between 10 and 12 weeks. Pretreatment blood samples were not collected prior to study initiation. Water and food were available *ad libitum* throughout the acclimation and experimental periods. PCH was prepared in phosphate buffered saline (PBS) on each treatment day and was administered via oral gavage in a volume of 0.003 mL/kg body weight/administration. Dose levels were 0, 6.25, 12.5, or 25 mg/kg/day and exposure occurred on study Days 1 through 28 (*n* = 4 for controls and 8 per treatment group per time point). The top dose level used in the main study was based on a pilot dose range‐finding study that demonstrated that a dose of 50 mg/kg/day resulted in a body weight loss that approached what would be considered excessive toxicity and grossly affected testis weight (this aspect will be reported separately, manuscript in progress).

For the micronucleus assay, peripheral blood was collected 2 days after the end of the exposure period (referred to hereafter as 28 + 2d) from the same groups of animals euthanized the next day (i.e., 28 + 3d) for the *lacZ* and *Pig‐a* assays. Approximately 60 μL of peripheral blood was collected from the facial vein and immediately combined with 350 μL of anticoagulant from the MicroFlow^BASIC^ Kit (Rodent Fixed Blood; Litron Laboratories, Rochester, NY). Following MicroFlow^BASIC^ Kit instructions, anticoagulated blood samples were fixed with ice‐cold methanol and stored at −80°C. After 5 days, fixed blood samples were centrifuged, rinsed, and transferred to a long‐term storage solution. Coded specimens were shipped to Litron for analysis of micronucleated cell frequency as described below.

Peripheral blood and bone marrow for the *Pig‐a* and *lacZ* assays, respectively, were collected at 28 + 3d and 70 days (28 + 70d) after cessation of treatment. At each time point, animals were euthanized by cardiac puncture under isoflurane anesthesia. The femurs were removed and the bone marrow was flushed out in 1 mL of PBS, pelleted via brief centrifugation, resuspended in 100 μL of PBS and flash frozen in liquid nitrogen, and finally stored at −80°C. Following blood collection via cardiac puncture, 400 μL of blood was immediately transferred to a 0.5 mL K_2_‐EDTA‐coated microtainer obtained from Litron and maintained at 4°C until being placed in an ExactPak shipping container and shipped on ice overnight to Litron for analysis of *Pig‐a* mutant cell frequencies as described below.

### Micronucleated Reticulocytes: Sample Preparation, Data Acquisition

The frequency of reticulocytes (%RET), micronucleated immature, and mature (normochromatic) erythrocytes (MN‐RET and MN‐NCE, respectively) were determined for coded blood samples collected at the 28 + 2d time point. To prepare samples for flow cytometric analysis, blood was washed out of methanol fixative and incubated with antibodies and other reagents as specified in the *In Vivo* Mouse MicroFlow Kit manual. This methodology has been described in detail elsewhere (Dertinger et al. 2004). Kit‐supplied malaria‐infected erythrocytes served as biological standards and guided instrument settings on each day of analysis (Tometsko et al. 1993). Micronucleus frequencies were determined upon the acquisition of 20,000 CD71‐positive reticulocytes (RET) per blood sample. A BD FACSCalibur flow cytometer running CellQuest Pro v5.2 was used for these analyses.

### LacZ Mutation: Sample Preparation, Data Acquisition

*LacZ* mutant frequencies were determined on coded bone marrow samples collected at 28 + 3d and 28 + 70d. Bone marrow total genomic DNA was extracted according to Gingerich et al. (2014). Briefly, bone marrow was thawed, then 50 μL was digested in 5 mL lysis buffer (10 mM EDTA, 100 mM NaCl, 10 mM Tris–HCl, pH 7.6, 1% sodium dodecyl sulfate [w/v] and 1 mg/mL Proteinase K [Invitrogen, Burlington, Canada]), and incubated at 37°C overnight with gentle shaking. Genomic DNA was extracted using phenol/chloroform extraction procedure and was then stored at 4°C in 90 μL TE buffer (10 mM Tris pH 7.6, 0.1 mM EDTA).

The P‐Gal positive selection assay was used for determining *lacZ* mutant frequency in DNA samples as previously described (Gingerich et al. 2014). The recombinant λgt10 phage vector was transfected from genomic DNA using Packaging Extract kits then mixed with the host bacterium (*Escherichia coli lacZ* ^*−*^/*galE* ^*−*^), plated on medium containing 0.3% (w/v) P‐Gal and incubated overnight at 37°C. Mutant frequency was calculated as the ratio of mutant plaque forming units (pfu) to total pfu. The DNA from two mice (a control and one middle dose at 28 + 3d) did not pack well and produced no colonies; while two mice (one from the low dose and one from the middle dose) had to be euthanized before tissue collection at 28 + 70d because of rectal prolapse.

### Pig‐a Mutation: Sample Preparation, Data Acquisition

*Pig‐a* mutant phenotype cell frequencies were determined on coded blood samples collected at 28 + 3d and 28 + 70d. Methods for processing mouse blood for *Pig‐a* measurements have been described (Labash et al. 2016). As with previous reports, the frequency of mutant phenotype reticulocytes (RET^CD24‐^) as well as the frequency of mutant phenotype erythrocytes (RBC^CD24‐^) was determined for each sample. An Instrument Calibration Standard was generated on each day data acquisition occurred. These samples contained a high prevalence of mutant‐mimic cells and provided a means to define the location of GPI anchor‐deficient erythrocytes (Phonethepswath et al. 2010). A BD FACSCanto II flow cytometer running FACSDiva v6.1.1 was used for the *Pig‐a* analyses.

### Calculations, Statistical Analyses

The incidence of MN‐RET, MN‐NCE, and RET are expressed as frequency percent. The formulas used to calculate RET^CD24‐^ and RBC^CD24‐^ frequencies based on pre‐ and post‐immunomagnetic column data are described by Dertinger et al. (2012) and the MutaFlow manual (www.litronlabs.com).

To evaluate the effect that PCH treatment may have had on %MN‐RET, %MN‐NCE, %RET, and RBC^CD24‐^ and RET^CD24‐^ frequencies relative to concurrent vehicle control mice, Dunnett's multiple comparison t‐tests were performed using JMP software's one‐way ANOVA platform (v12.0.1, SAS Institute, Cary, NC). With the exception of the %RET endpoint, each test was performed at the 5% level using a one‐tailed test to identify significant increases relative to vehicle control. In the case of %RET, the tests were two‐tailed in order to determine whether a significant treatment‐related increase or decrease occurred. Also, note that as similar frequencies were observed for vehicle control mice across every endpoint and both time points, we pooled these data to achieve a larger negative control group size.

Prior to performing the parametric tests described above, Levene's tests were used to verify the equality of variances in the samples (*P* > 0.05; JMP's ANOVA platform). The following transformations were applied to fulfill this requirement: %MN‐RET and %MN‐NCE values were cube root transformed; and RBC^CD24‐^, RET^CD24‐^, and *lacZ* mutation frequencies were log10 transformed.

Pearson correlation coefficients were calculated for *lacZ* and *Pig‐a* mean mutant frequencies at the 28 + 3d and 28 + 70d time points (Microsoft Excel for Mac 2008, v12.3.6, Microsoft, Redmond, WA). This was done for both RBC^CD24‐^ *vs. lacZ* mutant frequencies, and RET^CD24‐^ *vs. lacZ* mutant frequencies.

## RESULTS

We evaluated the induction of MN, *Pig‐a*, and *lacZ* mutations in the hematopoietic system of MutaMouse males at different time points after cessation of a 28‐day exposure to PCH. No significant reduction in body weight was observed at the end of the 4 weeks of exposure, or at the later time point (data not shown).

**Figure 1:**
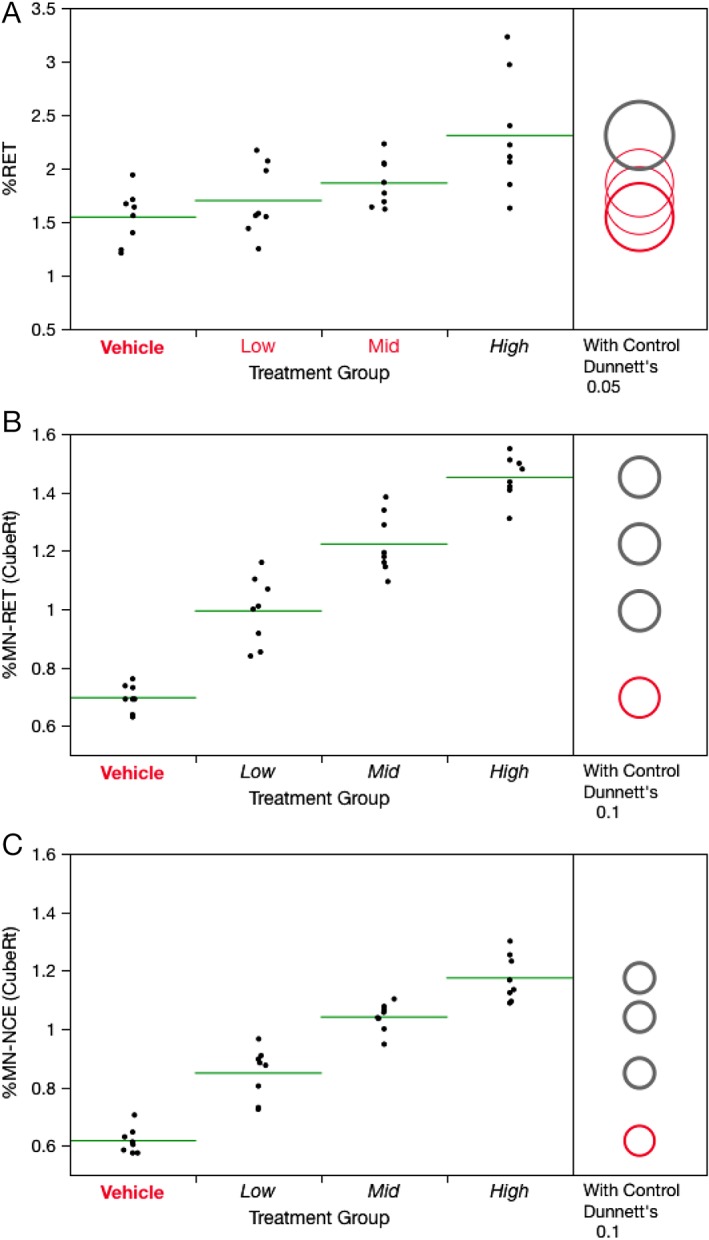
Frequency of Day 28 + 2d blood reticulocytes (**A**), micronucleated immature erythrocytes (**B**), and micronucleated mature erythrocytes (**C**) for each of four PCH treatment groups, where Low = 6.25, Mid = 12.5, and High = 25 mg/kg/day. Data for individual mice are shown, and group means appear as horizontal green lines. Dunnett's test results are shown to the far right of each graph, where statistically significant differences relative to the concurrent vehicle control group appear as italicized black text as opposed to red text, and by a gray circle as opposed to a red circle. Circles' diameters represent 95% confidence intervals (A) or 90% confidence intervals (B and C).

%RET were measured at 28 + 2d in conjunction with MN‐RET analyses. Slightly elevated %RET were observed, with a statistically significant increase evident for the high dose group (Fig. 1A). Higher %RET values that occur several days after cessation of treatment are generally indicative of treatment‐induced bone marrow toxicity, with a consequent period of enhanced erythropoiesis activity upon cessation of treatment (Dertinger et al. 2012). Robust, dose‐related MN induction was also observed for PCH‐exposed mice. Both MN‐RET and MN‐NCE frequencies exhibited statistically significant increases (*P* < 0.05), even at the low and middle dose levels that were not observed to appreciably affect erythropoiesis (Fig. 1B,C). For the high dose group, MN‐RET, and MN‐NCE frequencies exhibited mean fold increases of 9.1x and 6.8x, respectively.

**Figure 2:**
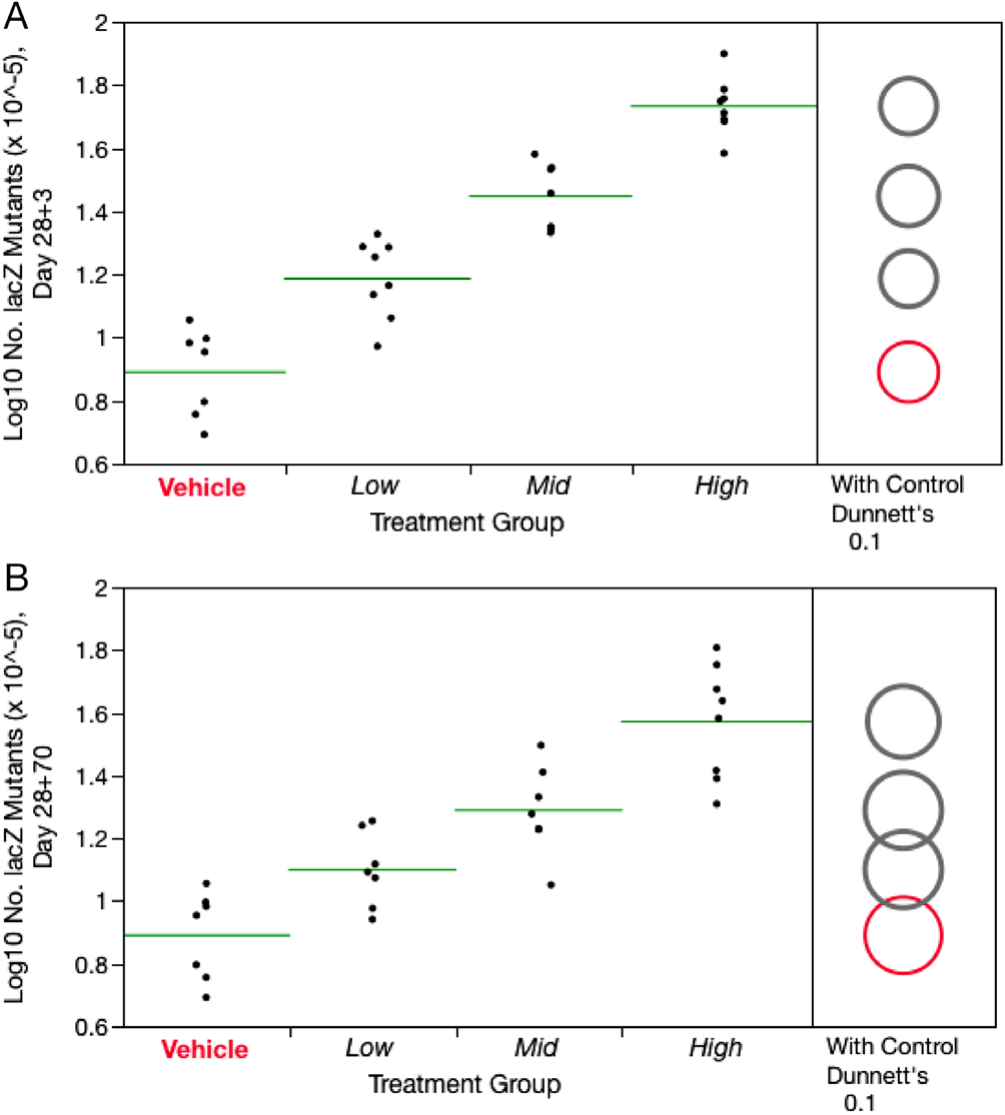
Frequency of Day 28 + 3d *lacZ* mutations (**A**) and Day 28 + 70d *lacZ* mutations (**B**) for each of four PCH treatment groups, where Low = 6.25, Mid = 12.5, and High = 25 mg/kg/day. Data for individual mice are shown, and group means appear as horizontal green lines. Dunnett's test results are shown to the far right of each graph, where statistically significant differences relative to the concurrent vehicle control group appear as italicized black text as opposed to red text, and by a gray circle as opposed to a red circle. Circles' diameters represent 90% confidence intervals.

As shown by Figure 2A, treatment with PCH for 28 consecutive days caused dose‐related increases in *lacZ* mutant frequencies at 28 + 3d with significant fold increases of 2.0‐, 3.5‐, and 6.8‐fold at the low, medium, and high doses (*P* < 0.05 for all doses), respectively. These effects were sustained at 28 + 70d, with fold increases of 1.6‐, 2.5‐, and 5.0‐ at the low, medium, and high dose (*P* < 0.05; Fig. 2B). Although the latter harvest time exhibited modestly lower mean PCH‐induced mutant frequencies, and within group variation was more pronounced, these results indicate that PCH induced mutagenicity in long‐lived bone marrow progenitor cells. Mean *lacZ* frequencies for each treatment condition and time point are provided in Supporting Information Table S1.

**Figure 3:**
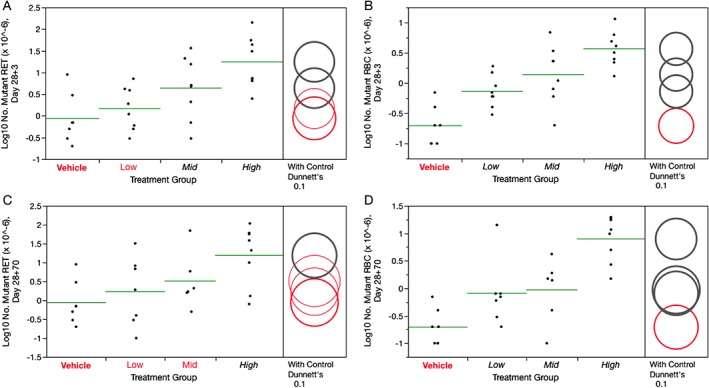
Frequency of 28 + 3d *Pig‐a* mutant reticulocytes (**A**) and *Pig‐a* mutant erythrocytes (**B**), as well as 28 + 70d *Pig‐a* mutant reticulocytes (**C**) and *Pig‐a* mutant erythrocytes (**D**) for each of four PCH treatment groups, where Low = 6.25, Mid = 12.5, and High = 25 mg/kg/day. Data for individual mice are shown, and group means appear as horizontal green lines. Dunnett's test results are shown to the far right of each graph, where statistically significant differences relative to the concurrent vehicle control group appear as italicized black text as opposed to red text, and by a gray circle as opposed to a red circle. Circles' diameters represent 90% confidence intervals.

RET^CD24‐^ frequencies were significantly increased in PCH‐treated mice on days 28 + 3d and 28 + 70d (Fig. 3A,C). RET^CD24‐^ responses were more variable at the later time point. As with the *lacZ* results, the fact that elevated RET^CD24‐^ frequencies were observed at the late time point suggests that some portion of the mutated cells arose from long‐lived progenitors with extended self‐renewal capacity. RBC^CD24‐^ frequencies also showed responses to PCH exposure, with statistically significant increases observed in all PCH treatment groups and at both time points (Fig. 3B,D). Note that the increase observed in the low dose group at 28 + 70d must be qualified to some extent because statistical significance is lost when the animal with the highest mutant frequency is removed. Even so, the result is not likely a scoring artifact as this animal also had the highest mutant RET value among the animals in the same group. The raw *Pig‐a* data and calculated frequencies, on a per mouse basis, are posted at the *Pig‐a In Vivo* Gene Mutation Assay Database, http://www.pharmacy.umaryland.edu/centers/cersi-files.

**Figure 4:**
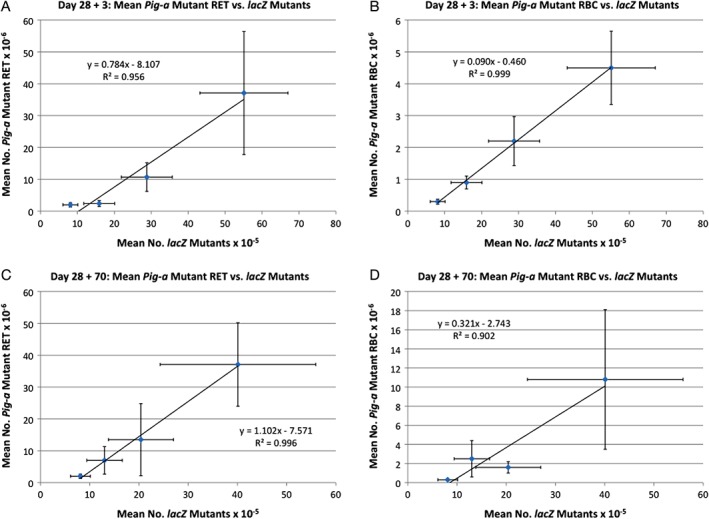
The mean frequency of *Pig‐a* mutant cells are graphed against the mean frequency of *lacZ* mutation for each of four dose groups and each of two time points, with Pearson correlation coefficients provided by an R^2^ value. Error bars represent one standard deviation. **A** = 28 + 3d results, where *Pig‐a* mutants are reticulocytes; **B** = 28 + 3d results, *Pig‐a* mutants are erythrocytes; **C** = 28 + 70d results, *Pig‐a* mutants are reticulocytes; and **D** = 28 + 70d results, *Pig‐a* mutants are erythrocytes.

Pearson correlation coefficients were derived for comparisons across the two mutation assays. At 28 + 3d, mean RET^CD24‐^ and RBC^CD24‐^ *vs. lacZ* frequencies showed particularly high correlations, with R^2^ values of 0.956 and 0.999, respectively (*P* < 0.05, Fig. 4A,B). At 28 + 70d, mean RET^CD24‐^ were also in good agreement with *lacZ* (R^2^ = 0.996, *P* < 0.05, Figure 4C), while the RBC^CD24‐^ correlation was slightly lower (R^2^ = 0.902, *P* < 0.05, Figure 4D).

## DISCUSSION

There is currently considerable pressure to transition the practice of toxicology toward computational and *in vitro* methodologies (Dix et al. 2007; Collins et al. 2008; Kavlock et al. 2009). In light of this, and given its predominately negative *in vitro* DNA‐damage profile, PCH made an interesting case study for evaluating multiple *in vivo* endpoints of genotoxicity—*lacZ* and *Pig‐a* mutation, and chromosomal damage in the form of MN. By concentrating on bone marrow and blood‐based assays, we focused on the hemotopoietic compartment and the notable hematological secondary cancers that have been described for PCH exposure (Levine and Bloomfield 1992). Furthermore, by using an experimental design that facilitated assessment of all three assays, this PCH study was performed in a manner that addresses resource and animal‐usage concerns that are in part driving the transition noted above.

In this study, PCH's *in vivo* genotoxic activity was readily apparent, as each of the endpoints investigated showed statistically significant effects at all of the doses tested. As also found by Suzuki et al. (1999), we observed potent clastogenic activity as evidenced by robust MN induction. Whereas Suzuki and colleagues failed to detect significant increases in *in vivo lacZ* mutations in bone marrow, liver, testis, spleen, kidney, and lung upon single administration of 50 mg PCH/kg, they did observe significant *lacZ* mutation induction at 150 mg/kg/day when administered for five consecutive days (cumulative dose: 750 mg/kg) particularly in those tissues that are prone to develop tumors following PCH exposure (i.e., lung, bone marrow, and spleen). In our experiments, we demonstrated that a strong mutagenic response in the bone marrow is occurring at much lower doses, as even at the lowest PCH dose studied (6.25 mg/kg/day, or a cumulative dose of 175 mg/kg) resulted in significant induction of bone marrow *lacZ* and blood *Pig‐a* mutation. These results show that PCH elicits a robust mutagenic response in the bone marrow even at doses that do not produce cytotoxicity. While Phonethepswath et al. (2013) also reported PCH‐induced *Pig‐a* mutation in mice, it is not productive comparing those results with data reported herein because the previous study utilized an early mouse blood labeling protocol that is now understood to systematically underestimate mutant cell frequency (Labash et al. 2016). Finally, Chen et al. (2019) recently reported strong induction of *Pig‐a* mutations following a 28‐day exposure regimen in rats using daily doses higher than those used here. Thus, in addition to its well‐known clastogenic activity, PCH is also an effective *in vivo* mutagen when recommended OECD test guideline experimental design considerations are followed.

One of the objectives of our study was to compare the mutagenic response observed with two reporter genes that are routinely used for assessing chemical mutagenesis. We found a very strong correlation (R^2^ ≥ 0.956) between *lacZ* mutant frequencies in bone marrow and *Pig‐a* mutant RET and RBC at the early time point (i.e., 28 + 3d). At the later time point (i.e., 28 + 70d), the correlation between the two assays was still very strong (R^2^ = 0.996) when comparing *lacZ* mutant frequencies with *Pig‐a* mutant RET. The correlation was slightly lower between mutant *lacZ* bone marrow cells and *Pig‐a* mutant RBC (R^2^ = 0.902), a result that reinforces expert recommendations to study both RET and RBC cohorts for *Pig‐a* mutation (Gollapudi et al. 2015). Overall, our mutation results are in agreement with those reported by Lemieux and collaborators (Lemieux et al. 2011) who observed a significant, and comparable, dose‐related increase in mutant cells with the *Pig‐a* and *lacZ* assays following exposure of MutaMouse males to benzo[a]pyrene, a strong mutagen that acts through a completely different mode of action compared to PCH. These results support the notion that both assays have a broad applicability domain and that they both are effective methods for evaluating gene mutation events.

The use of both gene mutation assays in an integrated test design represents a convenient means to address limitations that are generally ascribed to the two assays when used in isolation. The *lacZ* mutation assay is a well‐validated and regulatory‐accepted assay with an adopted OECD guideline that can be used for assessing mutagenicity in any tissue. A potential limitation of the assay is that it uses a bacterial gene that is highly methylated and not transcribed, and thus may not be fully representative of the susceptibility of endogenous genes to mutation induction (Lambert et al. 2005). It is also detecting almost exclusively point mutations (Beal et al. 2015). On the other hand, the *Pig‐a* gene is an endogenous gene and its inactivation can occur through a variety of mutational events including those that would not be detected by the *lacZ* assay. However, the *Pig‐a* assay can currently only be conducted in erythropoietic cells and it is challenging to sequence presumed mutant cells to characterize the types of lesions responsible for the mutant phenotype. It should also be noted that an OECD test guideline for the *Pig‐a* assay is currently under development, and the results reported herein add further support to these validation efforts.

In summary, our data agree with previous studies that found PCH exerts potent clastogenic activity. Furthermore, detection of its mutagenic potential is also readily observed when more careful analysis, particularly in terms of dosing protocol and harvest times, are followed. The advantage of combining the *lacZ*, *Pig‐a*, and MN endpoints is that it allows for a comprehensive assessment of multiple types of DNA lesions, a particularly important aspect when studying new chemical classes and/or when complex metabolism makes choosing a single *in vivo* genotoxicity endpoint difficult to choose *a priori*. By coupling the gene mutation assessments with MN analyses, it becomes possible to efficiently detect a chemical's mutagenic and chromosome damaging potential. Thus, when systemic exposure of a chemical and its reactive intermediate(s) can be verified, this approach represents an efficient and cost‐effective means of obtaining valuable safety data that includes mode of action information that has not traditionally been collected due to technical and cost considerations.

## AUTHOR CONTRIBUTIONS

F.M., C.Y., S.D. and C.M. designed these studies. F.M. and C.Y. secured funding for the study. C.M. executed the *lacZ* analyses, and prepared coded blood samples for shipment to Litron. C.M. and S.D.D. performed statistical analyses, and all of the authors contributed to data interpretation and writing the manuscript.

## DISCLOSURE

S.D.D is an employee of Litron Laboratories. Litron holds patents covering flow cytometric methods for scoring micronucleated erythrocytes and sells kits based on this technology (*In Vivo* MicroFlow®); Litron hold patents for scoring GPI anchor‐deficient erythrocytes and sells kits based on this technology (*In Vivo* MutaFlow®).

## Supporting information

Supplemental TABLE 1 *LacZ* mutant frequencies (MFs) in the bone marrow of MutaMouse males, 3 and 70 days after the end of a 28‐day exposure to procarbazine.
