# Supplementary material for: Integrated In Vivo Genotoxicity Assessment of Procarbazine Hydrochloride Demonstrates Induction of Pig‐a and LacZ Mutations, and Micronuclei, in MutaMouse Hematopoietic Cells
Source: Environ Mol Mutagen. 2019 Jan 18;60(6):505–12. doi: 10.1002/em.22271 (PMC6618172; doi:10.1002/em.22271)
Supplement: Supplementary file 1 — Supplemental TABLE 1 LacZ mutant frequencies (MFs) in the bone marrow of MutaMouse males, 3 and 70 days after the end of a 28‐day exposure to procarbazine. [file EM-60-505-s001.docx]

# Maurice et al. - Integrated *in vivo* genotoxicity assessment of procarbazine hydrochloride demonstrates induction of *Pig-a* and *LacZ* mutations, and micronuclei, in MutaMouse hematopoietic cells

**Supplemental Table 1.** *LacZ* mutant frequencies (MFs) in the bone marrow of MutaMouse males, three and seventy days after the end of a 28-day exposure to procarbazine.

| **Days after the end of exposure**  **(days)** | **Procarbazine Dose (mg/kg/day)** | **n** | **Number of Mutants** | **PFU^a^** | **Average^b^ MF (x10^-5^)** | **SD** | ***p*-value** |
| --- | --- | --- | --- | --- | --- | --- | --- |
| 3 | 0 | 7 | 105 | 1342082 | 8.1 | 2.5 | - |
|  | 6.25 | 8 | 207 | 1269375 | 15.9 | 4.2 | 0.0004* |
|  | 12.5 | 7 | 387 | 1315890 | 28.8 | 6.9 | <0.0001* |
|  | 25 | 8 | 904 | 1597855 | 55.1 | 11.9 | <0.0001* |
|  |  |  |  |  |  |  |  |
| 70 | 6.25 | 7 | 145 | 1147775 | 13 | 3.6 | 0.0906 |
|  | 12.5 | 7 | 254 | 1268711 | 20.4 | 6.6 | <0.0001* |
|  | 25 | 8 | 527 | 1281010 | 40.1 | 15.8 | <0.0001* |

^a^pfu: plaque forming units

^b^based on the arithmetic mean of individual animals

*p*-values correspond to one-sided Dunnett’s test results, and asterisks denote statistically significant increases compared to the vehicle control group.
